# Investigating the causal association of postpartum depression with cerebrovascular diseases and cognitive impairment: a Mendelian randomization study

**DOI:** 10.3389/fpsyt.2023.1196055

**Published:** 2023-06-22

**Authors:** Jia Li, Jinqiu Li, Lan Shen, Huan Wang, Tian Zheng, Ying Hui, Xiaoxuan Li

**Affiliations:** ^1^Department of Nursing, Zhuhai Campus of Zunyi Medical University, Guangdong, China; ^2^Department of Pediatrics, The Fifth Affiliated Hospital of Zunyi Medical University, Guangdong, China; ^3^Department of Obstetrics, Zhuhai Maternity and Child Health Care Hospital, Guangdong, China

**Keywords:** postpartum depression, cerebrovascular diseases, cognitive impairment, Mendelian randomization, causal association

## Abstract

**Background:**

Postpartum depression (PPD) is considered the most widespread puerperium complication. The associations of major depressive disorder with certain types of cerebrovascular diseases and cognitive function have been proposed, but the potential causal effects of PPD on these phenotypes are still unknown.

**Methods:**

A Mendelian randomization (MR) research design with various methods (e.g., inverse-variance weighted method and MR pleiotropy residual sum and outlier test) was adopted to establish a causal relationship between PPD with cerebrovascular diseases and cognitive impairment.

**Results:**

No causal relationship between PPD with carotid intima media thickness and cerebrovascular diseases (i.e., stroke, ischemic stroke, and cerebral aneurysm) was found. However, MR analyses indicated a causal association between PPD and decreased cognitive function (*P* = 3.55 × 10^−3^), which remained significant even after multiple comparison corrections using the Bonferroni method. Sensitivity analyses using weighted median and MR-Egger methods indicated a consistent direction of the association.

**Conclusion:**

The causal association between PPD and cognitive impairment indicates that cognitive impairment is a critical aspect of PPD and thus cannot be regarded as an epiphenomenon. Addressing cognitive impairment and lessening the symptoms associated with PPD independently play significant roles in the treatment of PPD.

## Introduction

Postpartum depression (PPD), also called postnatal depression, is a type of mental disorder related to childbirth that can affect both sexes (1). The Diagnostic and Statistical Manual of Mental Disorders (DSM-IV) defines PPD as an episode of major depression, with its onset happening within the first month of delivery (2). However, for practical purposes, the duration of onset can be extended to around the first year after delivery (3). PPD is considered the most widespread puerperium complication (4). Furthermore, fathers tend to experience PPD at similar rates compared with maternal depression (5). Various risk factors increase susceptibility to PPD, such as life and infant care stress, insomnia, prenatal depression, and inadequate social support, but PPD remains relatively underdiagnosed despite its high prevalence rates (6). The effects of PPD on infants have been well studied. For instance, PPD tends to make children more likely to develop emotional issues, such as depressive disorders and anxiety disorders (7). Furthermore, infant studies have reported that the negative effects of PPD, such as cognitive deficits, may transfer into childhood (8, 9). However, the effects of PPD on parents themselves are less investigated.

Several studies have aimed to investigate the effect of baseline depression on the late onset of vascular diseases. Through a meta-analysis, it was revealed that the risk of stroke was significantly higher among patients with depression, which is independent of other comorbidities, such as diabetes and hypertension (10). The existence of a dose–response relationship between depression and stroke has also been reported (11). In addition to cerebrovascular diseases, the status of cognitive impairment worsened with accumulating depressive episodes (12, 13). Information from available psychosocial literature has been insufficient to designate PPD as a distinct syndrome or as a sub-category of a major depressive disorder (MDD) (14). However, the clinical expertise and psychosocial data support the idea that a distinction of symptomatic patterns exists between MDD and PPD (15). Thus, based on the evidence, we hypothesized that PPD led to cerebrovascular diseases and cognitive impairment, which was tested by Mendelian randomization (MR) analyses. The MR method circumvents the issues of residual confounding and reverse causality in traditional epidemiological studies (16) and can be used to infer the causal relationship between exposure (i.e., PPD) and outcomes (i.e., cerebrovascular diseases and cognitive impairment).

## Methods

### Study design

**Figure 1:**
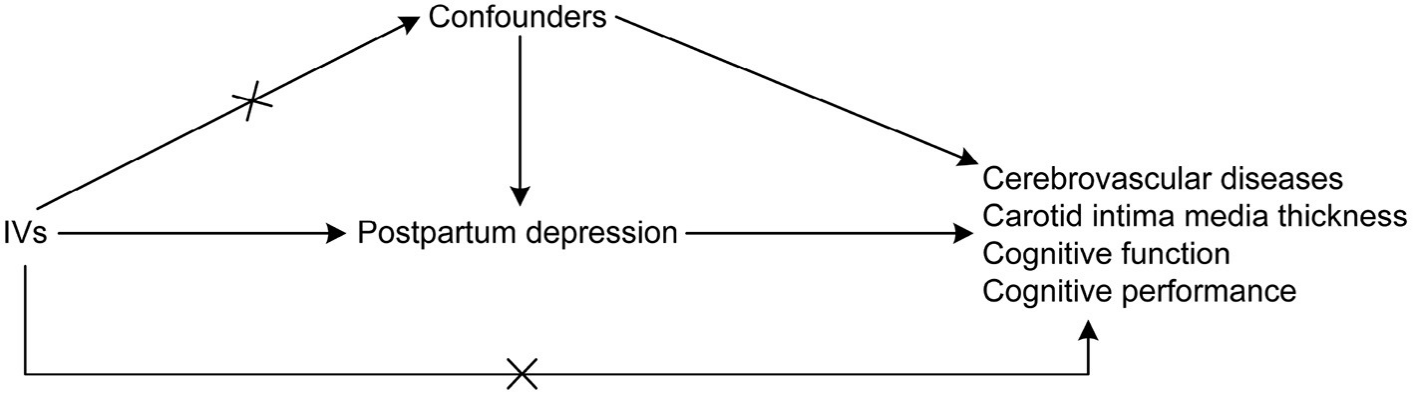
Directed acyclic graph showing the assumptions of MR analyses.

An MR research design was adopted to establish a causal relationship between PPD with cerebrovascular diseases and cognitive impairment. MR studies are based on the idea that alleles are randomly assigned during the meiotic phase of cell division, with conception as the basis of the natural experiment (17). In this design, the instrumental variables (IVs) used were genetic variations needed to derive the causal association of exposure with the outcome. Three assumptions were suggested for MR analyses (17) as follows: (1) there is a direct correlation between the single nucleotide polymorphisms (SNPs) utilized for PPD as IVs to the exposure; (2) the confounding variables cannot confound the IVs; and (3) the only causal pathway that links the IVs to the outcomes (i.e., cerebrovascular diseases and cognitive impairment) is through the PPD. The directed acyclic graph showing the assumptions of MR analyses is presented in Figure 1. In the MR analysis, the threshold of independent SNPs linked to the PPD as IVs was at a *P*-value of < 1 × 10^−5^. The F-statistics were also calculated to estimate the efficacy of the selected IVs.

### Data sources

The GWAS summary statistics data of PPD were obtained from FinnGen, where the status of delivery and International Classification of Diseases, Tenth Revision (ICD-10) code F32, F33, and F53.0 were used to define PPD (18). As very few SNPs were left if a genome-wide significance level of *P*-value (*P* < 5 × 10^−8^) was used to prepare the IVs for PPD, we used a P threshold of 1 × 10^−5^. The independence of the included SNPs as IVs was ensured by using a clumping method with a linkage disequilibrium threshold of r^2^ > 0.001 in a 10,000 kb window. The F-statistics of all the IVs, representing their ability to predict PPD, were calculated and found to be higher than 10 (Supplementary Table 1). The GWAS summary statistics data of stroke and ischemic stroke were obtained from a genome-wide association meta-analysis, focusing on stroke and its subtypes (19). The GWAS summary data of cerebral aneurysm were obtained from the analyses of UK Biobank data using a generalized linear mixed model-based genome-wide association tool, in which cerebral aneurysm was defined by the PheCode 433.5 (20). Summary statistics of carotid intima media thickness (cIMT) were also generated using data from UK Biobank (21). Cognitive function was estimated via a general cognitive function score, with a higher score representing better cognitive function and vice versa. This score was used in the GWAS analysis to generate the summary data (22). The GWAS summary statistics of cognitive performance were created by combining the analyses with general cognitive ability by the Cognitive Genomics Consortium (COGENT) and the analyses of cognitive performance by UK Biobank (23). More detailed information is summarized in Table 1.

**Table 1:** GWAS summary statistics used in the current study.

**Traits/diseases**	**Consortium**	**Sample size**
Postpartum depression	Finngen	249835
Stroke	NA	446696
Ischemic stroke	NA	440328
Cerebral aneurysm	UKB	456348
Carotid intima media thickness	UKB	45185
Cognitive function	Within family GWAS consortium	22593
Cognitive performance	NA	257841

GWAS, genome-wide association studies; UKB, UK Biobank; NA, not available.

### Statistical analysis

The summary statistics collected were aligned to the same single allele. We applied the inverse-variance weighted (IVW) MR method as the primary method to identify the potential associations between PDD and various phenotypes, such as cerebrovascular diseases (i.e., stroke, ischemic stroke, and cerebral aneurysm), cIMT (a measure for the diagnosis of carotid atherosclerotic vascular disease), cognitive function, and cognitive performance. The MR methods of MR-Egger, weighted median (WM), and MR pleiotropy residual sum and outlier (MR-PRESSO) were also applied for sensitivity analyses. The results of different MR analyses were shown on scatter plots and funnel plots, and the leave-one-out method was used to test the effect of individual SNPs by removing one SNP in turn. The MR-Egger regression was used to examine the genetic instruments for directional pleiotropy. Furthermore, the identification of heterogeneous results was accomplished through a Cochran-Q statistic test. A leave-one-out study with systematic elimination of one SNP at one time was also performed to examine the influence of the pleiotropic and/or marginal SNPs. Finally, the SNP outliers were identified using the MR-PRESSO program. The effects of PDD on binary phenotypes, such as cerebrovascular diseases, were presented as odds ratios (ORs) and 95% confidence intervals (CIs), while its effects on continuous variables, including cIMT, cognitive function, and cognitive performance, were presented as beta and 95% CIs. The Bonferroni correction was applied for multiple testing corrections. R software (version 4.2.1), together with its TwoSampleMR package, was applied in the current study (24).

## Results

**Figure 2:**
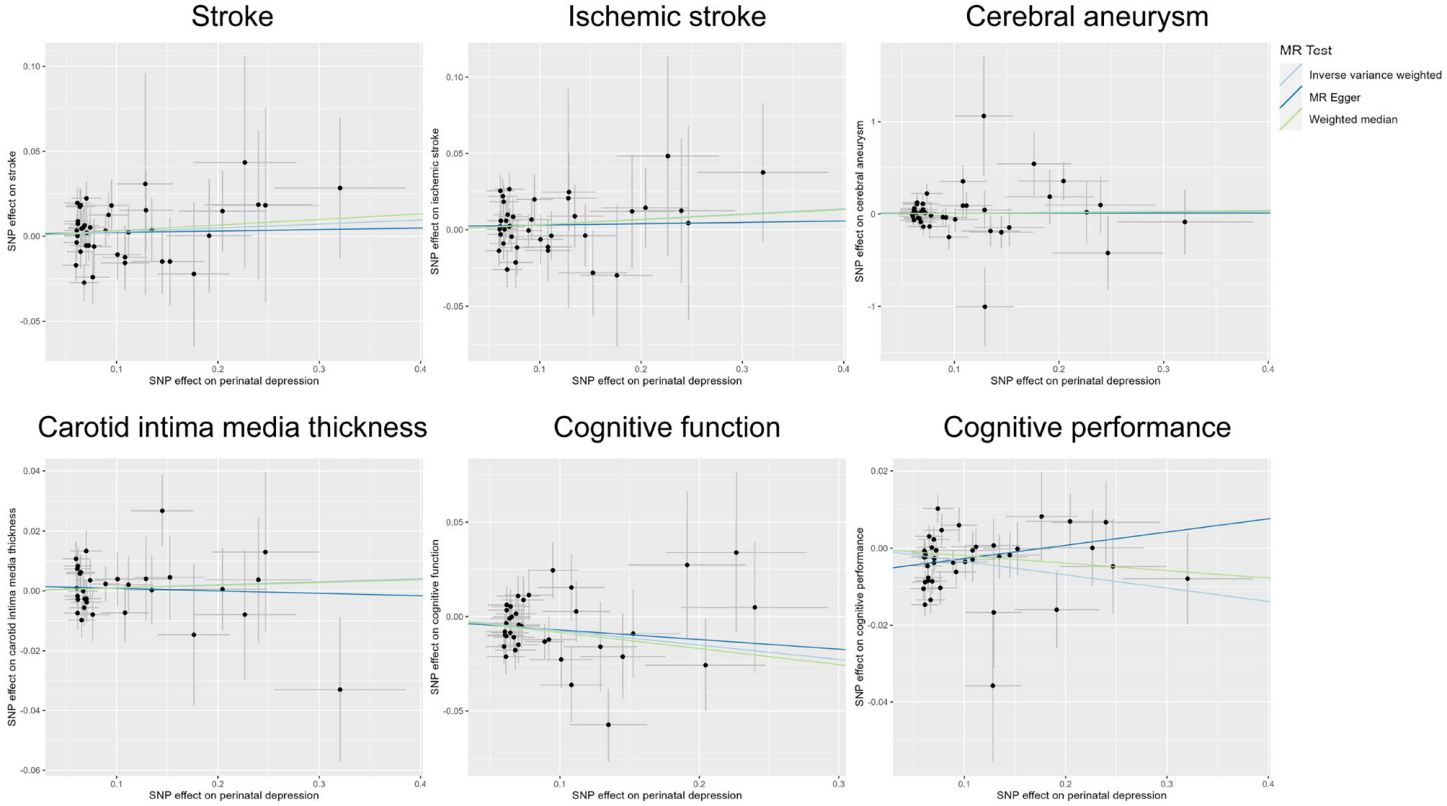
Scatter plots of MR analyses.

**Figure 3:**
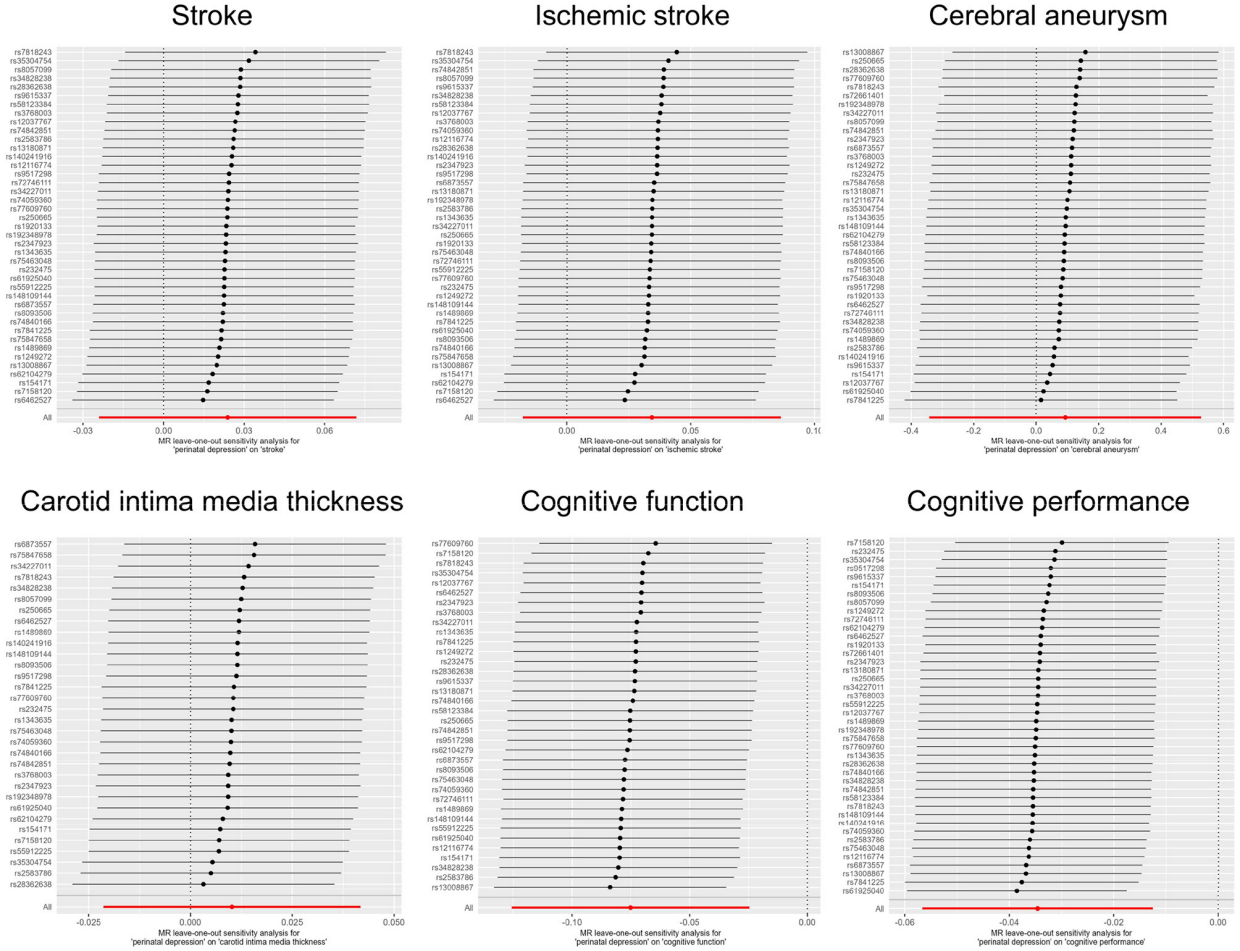
Leave-one-out plots of MR analyses.

Publicly available GWAS summary statistics data were collected (Table 1), and MR analyses were performed to test the potential causal association between PPD and a variety of phenotypes, including cerebrovascular diseases (i.e., stroke, ischemic stroke, and cerebral aneurysm), cIMT, cognitive function, and cognitive performance. The results of the MR analyses indicated that PPD has no causal relationship with cIMT and the cerebrovascular diseases included in this study (Table 2, Figure 2, Supplementary Figure 1, and Supplementary Table 2). However, MR analyses using the IVW method indicated that PPD was causally associated with both cognitive function (*P* = 3.55 × 10^−3^) and cognitive performance (*P* = 2.13 × 10^−3^), and these associations were still significant after multiple comparison corrections using the Bonferroni method (P threshold of 0.05/6 = 8.33 × 10^−3^). Thus, the result revealed that PPD led to a decreased level of cognitive function and cognitive performance, with the beta value of −0.075 (95% CI, −0.126-−0.025) and −0.035 (95% CI, −0.057-−0.013), respectively (Table 2, Figure 2, Supplementary Figure 1, and Supplementary Table 2). The IVs used in this study were good to predict PPD, as reflected by their F-statistics, with a mean of 22.4 and a range of 19.7–40.5 (Supplementary Table 1). Sensitivity analyses using the WM methods indicated a consistent direction of the association, but the direction of association analyzed using the MR-Egger method was consistent only for cognitive function but not cognitive performance (Table 2). Sensitivity analysis using the leave-one-out method indicated that the observed associations of PDD with the six phenotypes were not driven by single SNPs (Figure 3). A heterogeneity test revealed no significant heterogeneity, except for the analysis of the association between PDD and cognitive performance (Supplementary Table 3). Direct pleiotropy was also observed in the causal association of PDD with cognitive performance but not with cognitive function, as examined by the intercept of MR-Egger (Supplementary Table 4). In contrast, MR-PRESSO indicated a consistent result after removing outlier SNPs in the analysis (Supplementary Table 5).

**Table 2:** Results of MR study.

**Traits/diseases**	**Method**	**SNP number**	**OR/Beta**	**95% CI**	***P***
Stroke	IVW	41	1.024	(0.976-1.074)	3.30E-01
Stroke	MR Egger	41	1.009	(0.877-1.16)	9.03E-01
Stroke	WM	41	1.033	(0.965-1.106)	3.44E-01
Ischemic stroke	IVW	41	1.035	(0.982-1.09)	1.97E-01
Ischemic stroke	MR Egger	41	1.009	(0.866-1.175)	9.09E-01
Ischemic stroke	WM	41	1.034	(0.958-1.115)	3.92E-01
Cerebral aneurysm	IVW	41	1.098	(0.71-1.697)	6.75E-01
Cerebral aneurysm	MR Egger	41	1.005	(0.298-3.39)	9.93E-01
Cerebral aneurysm	WM	41	1.074	(0.602-1.916)	8.08E-01
Carotid intima media thickness	IVW	32	0.010	(−0.021-0.042)	5.26E-01
Carotid intima media thickness	MR Egger	32	−0.008	(−0.091-0.074)	8.47E-01
Carotid intima media thickness	WM	32	0.009	(−0.037-0.056)	6.95E-01
Cognitive function	IVW	36	−0.075	(−0.126-−0.025)	3.55E-03
Cognitive function	MR Egger	36	−0.050	(−0.217-0.116)	5.56E-01
Cognitive function	WM	36	−0.084	(−0.156-−0.012)	2.14E-02
Cognitive performance	IVW	42	−0.035	(−0.057-−0.013)	2.13E-03
Cognitive performance	MR Egger	42	0.034	(−0.022-0.091)	2.42E-01
Cognitive performance	WM	42	−0.019	(−0.041-0.002)	7.86E-02

SNP, single-nucleotide polymorphism; IVW, inverse variance weighted; WM, weighted median; OR, odds ratio; CI, confidence interval.

## Discussion

This study tested the potential causal relationship of PPD with various cerebrovascular diseases and cognitive impairment using GWAS summary statistics and MR designs. The analyzed results indicated that PPD was negatively associated with cognitive function, which was also supported by the MR sensitivity analyses using the MR-Egger and WM methods. Furthermore, the causal association between PPD and cognitive function was not caused by direct pleiotropy as determined by the MR-Egger intercept test. Thus, cognitive impairment is an essential aspect of PPD, and our study highlights the significance of recognizing the impact of PPD on cognitive function and addressing it when treating the condition.

Major depressive disorder (MDD) has been linked to vascular illnesses, as revealed by epidemiological studies, and several mechanisms have been established to show how depression and vascular illnesses exacerbate each other (25). Notable increments in the risk of coronary artery disease in patients presenting with baseline depressive disorders have been established (26). A dose–response relationship between the severity of depression and late-onset vascular disease has also been reported (25). There are several related theories through which depression reasonably heightens one's susceptibility to developing vascular problems. For instance, depression contributes to the changes in platelet function as well as the mechanism of clotting, which links to the atherogenic process (27). Furthermore, patients with depression were observed to have elevated levels of cortisol, which are intrinsically linked to the severity of coronary atherosclerosis (28). The administration of steroids has been shown to augment the levels of triglycerides and cholesterol, thereby contributing to the development of cardiovascular diseases (29). Moreover, depression is also linked to unhealthy lifestyles which exacerbate vascular diseases (25). However, our MR analyses indicated no causal association between PDD and a variety of cerebrovascular diseases, including stroke, ischemic stroke, and cerebral aneurysm, as well as cIMT, the indicator for carotid atherosclerotic vascular disease. This observation may highlight the differences between MDDs and PPD. Owing to the significant psychological, physiological, and hormonal changes during postpartum, it is reasonable to presume that depression would be exhibited in a distinctive manner (15). PPD can be initiated by sudden changes in sex hormones (30), and studies also revealed that women experience unique disorders with more diverse symptoms during PPD compared with the mental disorders that occurred in non-puerperal instances (15). Nonetheless, this research area requires further exploration because the main reason why PPD is often underdiagnosed is the insufficient knowledge of primary care specialists regarding the postpartum period.

MDD has been linked to a dysfunction in cognition, as reported by several epidemiological studies. Compromised cognition occurs in more than half of the patients diagnosed with depression (31). The DSM-IV-TR has a diagnostic criteria of an impaired ability for decision-making, concentration, or thought processing for major depressive episodes (31). For patients with late-life depression (LLD), cognitive impairment is usually diagnosed, and LLD may promote the development of dementia (32). Recently, a link between depression and issues with planning, problem-solving, and cognitive functioning has been reported. For instance, while patients with MDD have expected automatic processing performance, they are largely limited when executing tasks that require extended attention (33). Treatment with anti-depressants helped to improve cognitive outcomes in patients with MDD, although overall performance was still lower than that of healthy individuals (34). During the postpartum period, the cognitive functioning of women might become dysfunctional, and the decline in cognitive performance is often linked to the total time spent in labor and the use of labor analgesia (35). However, the association between PPD and cognitive functioning is far from being understood. Our study inferred the causal relationship of PPD with cognitive function and revealed that PDD could cause a decreased level of cognitive function.

Alteration of cognitive functions in patients with PDD could be caused by various endocrine factors such as the dysregulation of the hypothalamus–pituitary–gonadal axis, and an increase and decrease in progesterone and estradiol during pregnancy and after delivery, respectively, could be observed (36). In addition, a direct correlation exists between PPD symptoms and the severity of insomnia, a link that may also contribute to the changes in cognitive abilities (37). Decreases in cognitive function induced by PPD may also share common mechanisms by which MDD is associated with cognitive dysfunction. According to the global-diffuse hypothesis, people with depression normally tend to have a lower cognitive profile, representing an indication of a global-diffuse impairment in several cognitive domains (38). Since brain-derived neurotrophic factor plays a mediatory role in the dorsolateral prefrontal cortex plasticity and hippocampal forms such as the long-term potentiation, a reduction in its expression, as witnessed in chronic stress, could be attributed to depression-induced cognitive deficits (39). The involvement of limbic dopaminergic signaling has also been reported as a reason for dismal performance such as low level of sustained effort and reduced reward (40). Moreover, it was established that the decrease in white matter in LLD diminished emotion processing (41). The cognitive effort hypothesis indicates that patients with depression function normally in automatic tasks but struggle in effortful tasks (13). An analysis of cognitive function changes in patients with depression indicated that mood improvements were linked to developments in psychomotor speed, verbal fluency, and memory, whereas both executive and attentive functions remained unaltered during treatment (42). How PDD affects different aspects of cognitive functioning requires further investigation. Considering that genetics plays a controlling role in both cognitive ability and MDD (43), the usage of genome-wide association investigations to isolate the common genetic variants strongly linked with PPD and cognition is recommended. As a key feature of depression, cognitive dysfunction may interact with the onset of depression (44). Thus, it is also possible that cognitive impairment can reversely cause PPD, which is beyond the scope of this study.

In this study, the causal associations between PDD and cerebrovascular diseases and between PDD and cognitive impairment were investigated for the first time using an MR design with reduced changes in residual confounding and reverse causality, which represents the major strength of our study. Various MR methods were also applied in this study for sensitivity analyses, and potential direct pleiotropy and heterogeneity were systematically examined. Several limitations were also present in our study. First, we mainly used the GWAS summary statistics from Europe, resulting in a conclusion that is not generalizable to other races and ethnicities. Second, as a drawback of any MR study, the effect of potential direct pleiotropy could not be fully removed.

## Conclusion

This MR study revealed a causal association between PPD and cognitive impairment, indicating that cognitive impairment is a critical aspect of PPD and thus cannot be regarded as an epiphenomenon. Addressing cognitive impairment and lessening the symptoms associated with PPD independently play significant roles in the treatment of PPD.

## Data availability statement

The original contributions presented in the study are included in the article/Supplementary material, further inquiries can be directed to the corresponding author.

## Author contributions

JL designed the study. JL, JQL, and LS carried out the statistical analyses, and drafted the manuscript. HW, TZ, YH, and XXL reviewed the manuscript. All authors have read and approved the final manuscript.
