# Supplementary material for: Investigating the causal association of postpartum depression with cerebrovascular diseases and cognitive impairment: a Mendelian randomization study
Source: Front Psychiatry. 2023 Jun 22;14:1196055. doi: 10.3389/fpsyt.2023.1196055 (PMC10324563; doi:10.3389/fpsyt.2023.1196055)
Supplement: Supplementary file 1 [file Data_Sheet_1.docx]

*Supplementary Material*


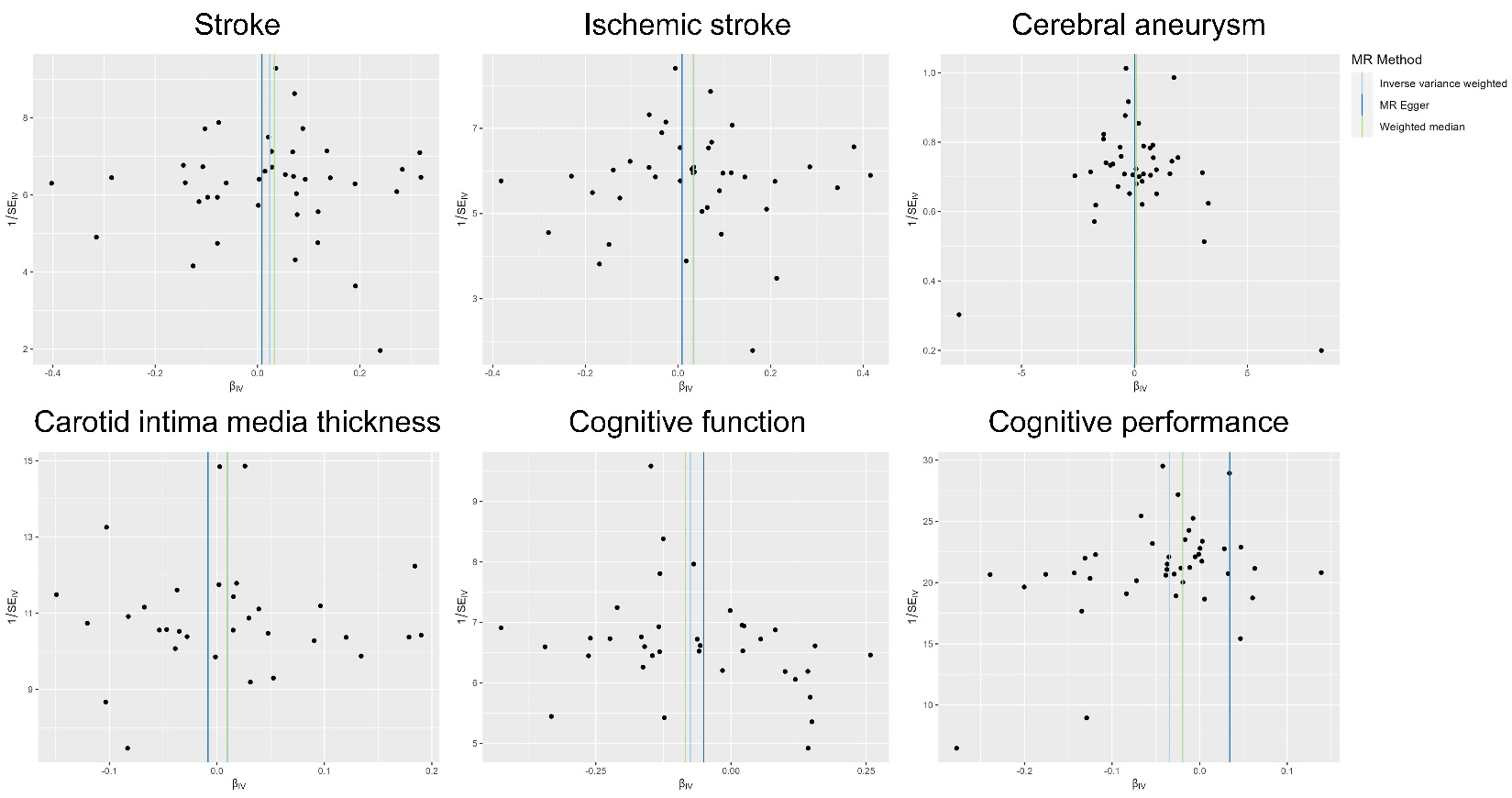


Supplementary Figure 1. Funnel plots of MR analyses

Supplementary Table 1. Instrumental variables for postpartum depression

| SNP | EA | OA | Beta | SE | P | F-Statistic |
| --- | --- | --- | --- | --- | --- | --- |
| rs12037767 | C | G | 0.108 | 0.023 | 1.49E-06 | 23.2 |
| rs1343635 | C | T | 0.061 | 0.013 | 4.68E-06 | 21.0 |
| rs3768003 | A | G | 0.101 | 0.023 | 8.10E-06 | 19.9 |
| rs72746111 | C | G | 0.191 | 0.042 | 5.12E-06 | 20.8 |
| rs12116774 | G | C | -0.078 | 0.017 | 5.14E-06 | 20.8 |
| rs13008867 | C | G | 0.095 | 0.018 | 1.43E-07 | 27.7 |
| rs77609760 | G | A | 0.135 | 0.028 | 1.19E-06 | 23.6 |
| rs28362638 | T | C | 0.145 | 0.031 | 2.32E-06 | 22.3 |
| rs1920133 | T | C | 0.128 | 0.028 | 4.13E-06 | 21.2 |
| rs35304754 | C | A | 0.060 | 0.013 | 6.82E-06 | 20.2 |
| rs154171 | C | T | -0.065 | 0.014 | 4.73E-06 | 20.9 |
| rs250665 | C | A | 0.071 | 0.015 | 4.74E-06 | 20.9 |
| rs13180871 | G | C | -0.061 | 0.013 | 5.20E-06 | 20.8 |
| rs6873557 | G | A | 0.065 | 0.014 | 1.61E-06 | 23.0 |
| rs2347923 | A | C | -0.089 | 0.014 | 1.99E-10 | 40.5 |
| rs6462527 | A | G | -0.070 | 0.014 | 1.17E-06 | 23.6 |
| rs74842851 | C | A | 0.153 | 0.034 | 5.66E-06 | 20.6 |
| rs7818243 | C | T | 0.068 | 0.015 | 3.15E-06 | 21.7 |
| rs2583786 | G | T | -0.070 | 0.015 | 5.98E-06 | 20.5 |
| rs7841225 | T | C | -0.205 | 0.043 | 2.52E-06 | 22.1 |
| rs74840166 | C | T | 0.129 | 0.027 | 1.16E-06 | 23.6 |
| rs75463048 | G | A | -0.240 | 0.053 | 5.63E-06 | 20.6 |
| rs1249272 | G | C | -0.092 | 0.020 | 3.29E-06 | 21.6 |
| rs61925040 | G | A | -0.074 | 0.015 | 1.13E-06 | 23.7 |
| rs192348978 | T | C | -0.247 | 0.053 | 2.82E-06 | 21.9 |
| rs34828238 | T | C | 0.108 | 0.024 | 5.70E-06 | 20.6 |
| rs9517298 | T | C | 0.062 | 0.013 | 4.84E-06 | 20.9 |
| rs72661401 | T | C | -0.129 | 0.028 | 3.91E-06 | 21.3 |
| rs7158120 | C | T | -0.061 | 0.014 | 8.43E-06 | 19.8 |
| rs34227011 | T | C | 0.062 | 0.013 | 3.83E-06 | 21.4 |
| rs74059360 | C | T | -0.112 | 0.024 | 4.56E-06 | 21.0 |
| rs1489869 | C | T | -0.062 | 0.013 | 3.33E-06 | 21.6 |
| rs58123384 | A | T | 0.072 | 0.014 | 9.13E-08 | 28.6 |
| rs75847658 | T | C | -0.321 | 0.065 | 7.16E-07 | 24.6 |
| rs8057099 | A | G | -0.077 | 0.017 | 6.60E-06 | 20.3 |
| rs148109144 | T | C | -0.227 | 0.051 | 7.50E-06 | 20.1 |
| rs55912225 | A | G | 0.062 | 0.014 | 5.66E-06 | 20.6 |
| rs8093506 | G | A | -0.069 | 0.014 | 1.19E-06 | 23.6 |
| rs62104279 | T | C | 0.064 | 0.014 | 8.89E-06 | 19.7 |
| rs140241916 | C | T | -0.176 | 0.036 | 7.22E-07 | 24.6 |
| rs232475 | A | G | 0.067 | 0.015 | 5.86E-06 | 20.5 |
| rs9615337 | G | C | 0.064 | 0.013 | 1.47E-06 | 23.2 |
| Abbreviation: SNP: single-nucleotide polymorphism; EA: effect allele; OA: other allele; SE: standard error. | | | | | | |

Supplementary Table 2. The associations between IVs and outcomes

| Traits/Diseases | SNP | EA | OA | Beta | SE | P |
| --- | --- | --- | --- | --- | --- | --- |
| Stroke | rs12037767 | C | G | -0.012 | 0.019 | 5.03E-01 |
|  | rs12116774 | G | C | 0.006 | 0.016 | 7.12E-01 |
|  | rs1249272 | G | C | -0.013 | 0.013 | 3.29E-01 |
|  | rs13008867 | C | G | 0.018 | 0.015 | 2.31E-01 |
|  | rs13180871 | G | C | 0.004 | 0.010 | 7.03E-01 |
|  | rs1343635 | C | T | 0.003 | 0.009 | 7.20E-01 |
|  | rs140241916 | C | T | 0.022 | 0.042 | 6.00E-01 |
|  | rs148109144 | T | C | -0.043 | 0.062 | 4.86E-01 |
|  | rs1489869 | C | T | -0.009 | 0.010 | 3.56E-01 |
|  | rs154171 | C | T | -0.018 | 0.010 | 5.98E-02 |
|  | rs1920133 | T | C | 0.031 | 0.065 | 6.37E-01 |
|  | rs192348978 | T | C | -0.018 | 0.057 | 7.50E-01 |
|  | rs232475 | A | G | 0.005 | 0.011 | 6.42E-01 |
|  | rs2347923 | A | C | -0.003 | 0.010 | 7.37E-01 |
|  | rs250665 | C | A | 0.002 | 0.011 | 8.49E-01 |
|  | rs2583786 | G | T | 0.006 | 0.012 | 6.41E-01 |
|  | rs28362638 | T | C | -0.015 | 0.019 | 4.27E-01 |
|  | rs34227011 | T | C | 0.001 | 0.009 | 9.22E-01 |
|  | rs34828238 | T | C | -0.016 | 0.016 | 3.26E-01 |
|  | rs35304754 | C | A | -0.017 | 0.009 | 6.55E-02 |
|  | rs3768003 | A | G | -0.011 | 0.015 | 4.72E-01 |
|  | rs55912225 | A | G | 0.007 | 0.013 | 5.72E-01 |
|  | rs58123384 | A | T | -0.006 | 0.009 | 5.50E-01 |
|  | rs61925040 | G | A | -0.005 | 0.011 | 6.47E-01 |
|  | rs62104279 | T | C | 0.017 | 0.011 | 9.88E-02 |
|  | rs6462527 | A | G | -0.022 | 0.010 | 2.38E-02 |
|  | rs6873557 | G | A | 0.005 | 0.009 | 6.24E-01 |
|  | rs7158120 | C | T | -0.020 | 0.010 | 4.01E-02 |
|  | rs72746111 | C | G | 0.000 | 0.033 | 9.94E-01 |
|  | rs74059360 | C | T | -0.002 | 0.015 | 8.71E-01 |
|  | rs74840166 | C | T | 0.015 | 0.023 | 5.10E-01 |
|  | rs74842851 | C | A | -0.015 | 0.026 | 5.64E-01 |
|  | rs75463048 | G | A | -0.019 | 0.044 | 6.71E-01 |
|  | rs75847658 | T | C | -0.028 | 0.042 | 4.93E-01 |
|  | rs77609760 | G | A | 0.004 | 0.019 | 8.46E-01 |
|  | rs7818243 | C | T | -0.027 | 0.011 | 1.13E-02 |
|  | rs7841225 | T | C | -0.015 | 0.024 | 5.33E-01 |
|  | rs8057099 | A | G | 0.024 | 0.016 | 1.22E-01 |
|  | rs8093506 | G | A | -0.006 | 0.011 | 5.50E-01 |
|  | rs9517298 | T | C | 0.000 | 0.010 | 9.86E-01 |
|  | rs9615337 | G | C | -0.009 | 0.010 | 3.73E-01 |
| Ischemic stroke | rs12037767 | C | G | -0.014 | 0.020 | 5.03E-01 |
|  | rs12116774 | G | C | 0.012 | 0.018 | 5.23E-01 |
|  | rs1249272 | G | C | -0.007 | 0.014 | 6.28E-01 |
|  | rs13008867 | C | G | 0.020 | 0.017 | 2.28E-01 |
|  | rs13180871 | G | C | 0.000 | 0.011 | 9.75E-01 |
|  | rs1343635 | C | T | 0.002 | 0.010 | 8.45E-01 |
|  | rs140241916 | C | T | 0.030 | 0.046 | 5.18E-01 |
|  | rs148109144 | T | C | -0.048 | 0.065 | 4.59E-01 |
|  | rs1489869 | C | T | -0.006 | 0.010 | 5.60E-01 |
|  | rs154171 | C | T | -0.018 | 0.011 | 8.27E-02 |
|  | rs1920133 | T | C | 0.021 | 0.072 | 7.73E-01 |
|  | rs192348978 | T | C | -0.004 | 0.064 | 9.45E-01 |
|  | rs232475 | A | G | 0.006 | 0.012 | 6.18E-01 |
|  | rs2347923 | A | C | 0.001 | 0.011 | 9.59E-01 |
|  | rs250665 | C | A | 0.003 | 0.012 | 8.32E-01 |
|  | rs2583786 | G | T | -0.002 | 0.012 | 8.54E-01 |
|  | rs28362638 | T | C | -0.004 | 0.020 | 8.53E-01 |
|  | rs34227011 | T | C | 0.002 | 0.010 | 8.38E-01 |
|  | rs34828238 | T | C | -0.011 | 0.017 | 5.20E-01 |
|  | rs35304754 | C | A | -0.014 | 0.010 | 1.73E-01 |
|  | rs3768003 | A | G | -0.006 | 0.017 | 7.03E-01 |
|  | rs55912225 | A | G | 0.006 | 0.014 | 6.73E-01 |
|  | rs58123384 | A | T | -0.005 | 0.010 | 6.44E-01 |
|  | rs61925040 | G | A | -0.009 | 0.012 | 4.91E-01 |
|  | rs62104279 | T | C | 0.022 | 0.011 | 5.35E-02 |
|  | rs6462527 | A | G | -0.027 | 0.011 | 1.25E-02 |
|  | rs6873557 | G | A | 0.000 | 0.010 | 9.78E-01 |
|  | rs7158120 | C | T | -0.026 | 0.010 | 1.45E-02 |
|  | rs72746111 | C | G | 0.012 | 0.037 | 7.46E-01 |
|  | rs74059360 | C | T | 0.004 | 0.016 | 8.11E-01 |
|  | rs74840166 | C | T | 0.025 | 0.025 | 3.27E-01 |
|  | rs74842851 | C | A | -0.028 | 0.028 | 3.11E-01 |
|  | rs75463048 | G | A | -0.013 | 0.048 | 7.92E-01 |
|  | rs75847658 | T | C | -0.038 | 0.045 | 4.07E-01 |
|  | rs77609760 | G | A | 0.009 | 0.021 | 6.65E-01 |
|  | rs7818243 | C | T | -0.026 | 0.012 | 2.81E-02 |
|  | rs7841225 | T | C | -0.014 | 0.026 | 5.79E-01 |
|  | rs8057099 | A | G | 0.021 | 0.017 | 2.03E-01 |
|  | rs8093506 | G | A | -0.010 | 0.012 | 3.95E-01 |
|  | rs9517298 | T | C | -0.003 | 0.011 | 7.78E-01 |
|  | rs9615337 | G | C | -0.009 | 0.011 | 3.98E-01 |
| Cerebral aneurysm | rs12037767 | C | G | 0.354 | 0.174 | 4.13E-02 |
|  | rs12116774 | G | C | 0.017 | 0.119 | 8.88E-01 |
|  | rs1249272 | G | C | 0.038 | 0.105 | 7.21E-01 |
|  | rs13008867 | C | G | -0.251 | 0.135 | 6.35E-02 |
|  | rs13180871 | G | C | 0.027 | 0.086 | 7.54E-01 |
|  | rs1343635 | C | T | 0.004 | 0.084 | 9.59E-01 |
|  | rs140241916 | C | T | -0.544 | 0.343 | 1.13E-01 |
|  | rs148109144 | T | C | -0.019 | 0.333 | 9.55E-01 |
|  | rs1489869 | C | T | -0.060 | 0.086 | 4.84E-01 |
|  | rs154171 | C | T | -0.125 | 0.086 | 1.46E-01 |
|  | rs1920133 | T | C | 1.062 | 0.643 | 9.86E-02 |
|  | rs192348978 | T | C | 0.423 | 0.399 | 2.89E-01 |
|  | rs232475 | A | G | -0.049 | 0.100 | 6.24E-01 |
|  | rs2347923 | A | C | 0.033 | 0.088 | 7.05E-01 |
|  | rs250665 | C | A | -0.137 | 0.099 | 1.65E-01 |
|  | rs2583786 | G | T | -0.110 | 0.099 | 2.65E-01 |
|  | rs28362638 | T | C | -0.197 | 0.176 | 2.63E-01 |
|  | rs34227011 | T | C | -0.065 | 0.084 | 4.35E-01 |
|  | rs34828238 | T | C | 0.090 | 0.143 | 5.29E-01 |
|  | rs35304754 | C | A | -0.004 | 0.085 | 9.58E-01 |
|  | rs3768003 | A | G | -0.059 | 0.133 | 6.57E-01 |
|  | rs58123384 | A | T | 0.014 | 0.085 | 8.73E-01 |
|  | rs61925040 | G | A | -0.222 | 0.104 | 3.22E-02 |
|  | rs62104279 | T | C | 0.013 | 0.091 | 8.86E-01 |
|  | rs6462527 | A | G | -0.049 | 0.090 | 5.88E-01 |
|  | rs6873557 | G | A | -0.041 | 0.083 | 6.20E-01 |
|  | rs7158120 | C | T | -0.024 | 0.087 | 7.78E-01 |
|  | rs72661401 | T | C | 1.003 | 0.426 | 1.87E-02 |
|  | rs72746111 | C | G | 0.187 | 0.294 | 5.24E-01 |
|  | rs74059360 | C | T | -0.091 | 0.141 | 5.18E-01 |
|  | rs74840166 | C | T | 0.044 | 0.208 | 8.31E-01 |
|  | rs74842851 | C | A | -0.146 | 0.207 | 4.82E-01 |
|  | rs75463048 | G | A | -0.098 | 0.304 | 7.48E-01 |
|  | rs75847658 | T | C | 0.086 | 0.350 | 8.06E-01 |
|  | rs77609760 | G | A | -0.184 | 0.167 | 2.69E-01 |
|  | rs7818243 | C | T | -0.086 | 0.092 | 3.50E-01 |
|  | rs7841225 | T | C | -0.357 | 0.207 | 8.52E-02 |
|  | rs8057099 | A | G | 0.136 | 0.134 | 3.12E-01 |
|  | rs8093506 | G | A | -0.023 | 0.100 | 8.14E-01 |
|  | rs9517298 | T | C | 0.044 | 0.087 | 6.15E-01 |
|  | rs9615337 | G | C | 0.107 | 0.086 | 2.17E-01 |
| Carotid intima media thickness | rs1343635 | C | T | 0.001 | 0.006 | 8.73E-01 |
|  | rs140241916 | C | T | 0.015 | 0.024 | 5.35E-01 |
|  | rs148109144 | T | C | 0.008 | 0.022 | 7.13E-01 |
|  | rs1489869 | C | T | 0.003 | 0.006 | 6.21E-01 |
|  | rs154171 | C | T | -0.006 | 0.006 | 2.81E-01 |
|  | rs192348978 | T | C | -0.013 | 0.027 | 6.25E-01 |
|  | rs232475 | A | G | 0.000 | 0.007 | 9.90E-01 |
|  | rs2347923 | A | C | -0.002 | 0.006 | 6.98E-01 |
|  | rs250665 | C | A | -0.004 | 0.007 | 5.71E-01 |
|  | rs2583786 | G | T | -0.013 | 0.007 | 4.75E-02 |
|  | rs28362638 | T | C | 0.027 | 0.012 | 2.42E-02 |
|  | rs34227011 | T | C | -0.007 | 0.006 | 1.95E-01 |
|  | rs34828238 | T | C | -0.007 | 0.010 | 4.52E-01 |
|  | rs35304754 | C | A | 0.011 | 0.006 | 6.37E-02 |
|  | rs3768003 | A | G | 0.004 | 0.009 | 6.65E-01 |
|  | rs55912225 | A | G | 0.008 | 0.006 | 1.85E-01 |
|  | rs61925040 | G | A | -0.004 | 0.007 | 6.18E-01 |
|  | rs62104279 | T | C | 0.006 | 0.006 | 3.52E-01 |
|  | rs6462527 | A | G | 0.003 | 0.006 | 6.67E-01 |
|  | rs6873557 | G | A | -0.010 | 0.006 | 8.64E-02 |
|  | rs7158120 | C | T | -0.007 | 0.006 | 2.12E-01 |
|  | rs74059360 | C | T | -0.002 | 0.009 | 8.29E-01 |
|  | rs74840166 | C | T | 0.004 | 0.014 | 7.74E-01 |
|  | rs74842851 | C | A | 0.005 | 0.014 | 7.46E-01 |
|  | rs75463048 | G | A | -0.004 | 0.021 | 8.60E-01 |
|  | rs75847658 | T | C | 0.033 | 0.024 | 1.73E-01 |
|  | rs77609760 | G | A | 0.000 | 0.011 | 9.82E-01 |
|  | rs7818243 | C | T | -0.006 | 0.006 | 3.69E-01 |
|  | rs7841225 | T | C | -0.001 | 0.014 | 9.68E-01 |
|  | rs8057099 | A | G | 0.008 | 0.009 | 3.70E-01 |
|  | rs8093506 | G | A | 0.003 | 0.007 | 6.97E-01 |
|  | rs9517298 | T | C | -0.002 | 0.006 | 7.73E-01 |
| Cognitive function | rs12037767 | C | G | -0.036 | 0.020 | 7.08E-02 |
|  | rs12116774 | G | C | -0.011 | 0.014 | 3.99E-01 |
|  | rs1249272 | G | C | 0.012 | 0.012 | 3.07E-01 |
|  | rs13008867 | C | G | 0.025 | 0.015 | 9.67E-02 |
|  | rs13180871 | G | C | 0.008 | 0.009 | 3.89E-01 |
|  | rs1343635 | C | T | -0.010 | 0.009 | 2.91E-01 |
|  | rs148109144 | T | C | -0.034 | 0.042 | 4.22E-01 |
|  | rs1489869 | C | T | -0.003 | 0.009 | 7.11E-01 |
|  | rs154171 | C | T | -0.005 | 0.009 | 5.71E-01 |
|  | rs232475 | A | G | -0.011 | 0.011 | 3.09E-01 |
|  | rs2347923 | A | C | 0.013 | 0.009 | 1.55E-01 |
|  | rs250665 | C | A | -0.004 | 0.011 | 6.77E-01 |
|  | rs2583786 | G | T | -0.011 | 0.011 | 3.02E-01 |
|  | rs28362638 | T | C | -0.021 | 0.023 | 3.48E-01 |
|  | rs34227011 | T | C | -0.010 | 0.009 | 2.64E-01 |
|  | rs34828238 | T | C | 0.015 | 0.018 | 3.78E-01 |
|  | rs35304754 | C | A | -0.016 | 0.009 | 8.99E-02 |
|  | rs3768003 | A | G | -0.023 | 0.015 | 1.32E-01 |
|  | rs55912225 | A | G | 0.006 | 0.010 | 5.40E-01 |
|  | rs58123384 | A | T | -0.005 | 0.009 | 5.84E-01 |
|  | rs61925040 | G | A | -0.009 | 0.012 | 4.74E-01 |
|  | rs62104279 | T | C | -0.001 | 0.010 | 9.23E-01 |
|  | rs6462527 | A | G | 0.015 | 0.010 | 1.27E-01 |
|  | rs6873557 | G | A | 0.000 | 0.009 | 9.89E-01 |
|  | rs7158120 | C | T | 0.021 | 0.009 | 2.36E-02 |
|  | rs72746111 | C | G | 0.027 | 0.039 | 4.83E-01 |
|  | rs74059360 | C | T | -0.003 | 0.016 | 8.66E-01 |
|  | rs74840166 | C | T | -0.016 | 0.024 | 5.05E-01 |
|  | rs74842851 | C | A | -0.009 | 0.023 | 7.02E-01 |
|  | rs75463048 | G | A | -0.005 | 0.035 | 8.87E-01 |
|  | rs77609760 | G | A | -0.057 | 0.020 | 3.29E-03 |
|  | rs7818243 | C | T | -0.018 | 0.010 | 7.82E-02 |
|  | rs7841225 | T | C | 0.026 | 0.024 | 2.93E-01 |
|  | rs8093506 | G | A | -0.002 | 0.011 | 8.83E-01 |
|  | rs9517298 | T | C | -0.004 | 0.009 | 7.05E-01 |
|  | rs9615337 | G | C | -0.009 | 0.009 | 3.57E-01 |
| Cognitive performance | rs12037767 | C | G | -0.003 | 0.006 | 6.09E-01 |
|  | rs12116774 | G | C | -0.005 | 0.004 | 2.58E-01 |
|  | rs1249272 | G | C | 0.006 | 0.004 | 8.88E-02 |
|  | rs13008867 | C | G | 0.006 | 0.004 | 1.84E-01 |
|  | rs13180871 | G | C | 0.002 | 0.003 | 4.27E-01 |
|  | rs1343635 | C | T | -0.001 | 0.003 | 8.10E-01 |
|  | rs140241916 | C | T | -0.008 | 0.011 | 4.72E-01 |
|  | rs148109144 | T | C | 0.000 | 0.010 | 9.93E-01 |
|  | rs1489869 | C | T | 0.001 | 0.003 | 6.51E-01 |
|  | rs154171 | C | T | 0.008 | 0.003 | 8.11E-03 |
|  | rs1920133 | T | C | -0.036 | 0.020 | 7.24E-02 |
|  | rs192348978 | T | C | 0.005 | 0.012 | 7.01E-01 |
|  | rs232475 | A | G | -0.013 | 0.003 | 8.34E-05 |
|  | rs2347923 | A | C | 0.004 | 0.003 | 2.13E-01 |
|  | rs250665 | C | A | -0.003 | 0.003 | 4.31E-01 |
|  | rs2583786 | G | T | -0.002 | 0.003 | 5.00E-01 |
|  | rs28362638 | T | C | -0.002 | 0.006 | 7.65E-01 |
|  | rs34227011 | T | C | -0.002 | 0.003 | 4.25E-01 |
|  | rs34828238 | T | C | -0.001 | 0.005 | 9.10E-01 |
|  | rs35304754 | C | A | -0.011 | 0.003 | 2.79E-04 |
|  | rs3768003 | A | G | -0.004 | 0.005 | 4.37E-01 |
|  | rs55912225 | A | G | -0.002 | 0.003 | 5.47E-01 |
|  | rs58123384 | A | T | -0.001 | 0.003 | 8.48E-01 |
|  | rs61925040 | G | A | -0.010 | 0.004 | 3.86E-03 |
|  | rs62104279 | T | C | -0.005 | 0.003 | 1.45E-01 |
|  | rs6462527 | A | G | 0.004 | 0.003 | 2.11E-01 |
|  | rs6873557 | G | A | 0.003 | 0.003 | 2.81E-01 |
|  | rs7158120 | C | T | 0.015 | 0.003 | 7.40E-07 |
|  | rs72661401 | T | C | 0.017 | 0.014 | 2.48E-01 |
|  | rs72746111 | C | G | -0.016 | 0.010 | 1.11E-01 |
|  | rs74059360 | C | T | 0.000 | 0.005 | 9.44E-01 |
|  | rs74840166 | C | T | 0.001 | 0.007 | 9.17E-01 |
|  | rs74842851 | C | A | 0.000 | 0.007 | 9.81E-01 |
|  | rs75463048 | G | A | -0.007 | 0.011 | 5.22E-01 |
|  | rs75847658 | T | C | 0.008 | 0.012 | 5.02E-01 |
|  | rs77609760 | G | A | -0.002 | 0.006 | 6.95E-01 |
|  | rs7818243 | C | T | 0.000 | 0.003 | 9.56E-01 |
|  | rs7841225 | T | C | -0.007 | 0.007 | 3.25E-01 |
|  | rs8057099 | A | G | 0.010 | 0.004 | 1.73E-02 |
|  | rs8093506 | G | A | 0.009 | 0.003 | 1.09E-02 |
|  | rs9517298 | T | C | -0.009 | 0.003 | 2.93E-03 |
|  | rs9615337 | G | C | -0.008 | 0.003 | 3.95E-03 |
| Abbreviation: SNP: single-nucleotide polymorphism; EA: effect allele; OA: other allele; SE: standard error | | | | | | |

Supplementary Table 3. Tests of heterogeneity

| Traits/Diseases | Q | Q_df | P |
| --- | --- | --- | --- |
| Stroke | 38.89 | 40 | 5.20E-01 |
| Ischemic stroke | 36.32 | 40 | 6.37E-01 |
| Cerebral aneurysm | 42.87 | 40 | 3.49E-01 |
| Carotid intima media thickness | 28.66 | 31 | 5.87E-01 |
| Cognitive function | 37.63 | 35 | 3.50E-01 |
| Cognitive performance | 100.70 | 41 | 6.22E-07 |

Supplementary Table 4. Tests of pleiotropy by MR-Egger

| Traits/Diseases | Egger_intercept | SE | P |
| --- | --- | --- | --- |
| Stroke | 0.001 | 0.006 | 8.24E-01 |
| Ischemic stroke | 0.002 | 0.006 | 7.31E-01 |
| Cerebral aneurysm | 0.008 | 0.051 | 8.80E-01 |
| Carotid intima media thickness | 0.002 | 0.003 | 6.40E-01 |
| Cognitive function | -0.002 | 0.007 | 7.62E-01 |
| Cognitive performance | -0.006 | 0.002 | 1.44E-02 |

Supplementary Table 5. MR-Presso test for the trait cognitive performance

| Main.MR.results.MR.Analysis | Main.MR.results.Causal.Estimate | Main.MR.results.Sd | Main.MR.results.T.stat | Main.MR.results.P.value |
| --- | --- | --- | --- | --- |
| Raw | -0.035 | 0.011 | -3.072 | 3.77E-03 |
| Outlier-corrected | -0.030 | 0.009 | -3.287 | 2.18E-03 |
